# Contributions of precipitation and temperature to the large scale geographic distribution of fleshy-fruited plant species: Growth form matters

**DOI:** 10.1038/s41598-018-35436-x

**Published:** 2018-11-19

**Authors:** Yuan Zhao, Honglin Cao, Wubing Xu, Guoke Chen, Juyu Lian, Yanjun Du, Keping Ma

**Affiliations:** 1 South China Botanical Garden, Chinese Academy of Sciences, 510650 Guangzhou, China; 2 Institute of Tropical Agriculture and Forestry, Hainan University, 570228 Haikou, China; 3 State Key Laboratory of Vegetation and Environmental Change, Institute of Botany, Chinese Academy of Sciences, 100093 Beijing, China; 4 College of Urban and Environmental Sciences, Laboratory for Earth Surface Processes of the Ministry of Education, Peking University, 100871 Beijing, China

## Abstract

Fruit type, an important reproductive trait, is closely related to reproduction strategy, community dynamics and biotic interactions. However, limited research has explored the geographic distribution of fruit type and the underlying abiotic factors influencing this on a large scale. Here we aim to study large-scale distribution patterns of fleshy-fruited plant species and the most important environmental drivers for different growth forms in utilizing the fruit type and distribution data for over 27000 plant species in China. Results indicated that the proportion of fleshy-fruited species was higher in southeast China, and this pattern was consistent between different growth forms. Overall, the proportion of fleshy-fruited species was higher in wet, warm, and stable environments. Notably, mean annual precipitation had the greatest predictive contribution to woody fleshy-fruited species distributions, but mean annual temperature best predicted the herbaceous fleshy-fruited species distributions. We provide the first map of a large-scale distribution of fleshy-fruited plant species for different growth forms in the northern hemisphere and show that these geographic patterns are mainly determined by contrasting climatic gradients. Recognizing that climate factors have different relationships with different growth forms of fleshy-fruited species advances our knowledge about fruit type and environment. This work contributes to predictions of the global distribution of fleshy-fruited species under future climate change scenarios and provides a reference for continued research on the complex interactions between plants, frugivores and the environment.

## Introduction

Functional biogeography is an emerging field that focuses on the geographical distribution of trait diversity and its relationship with environmental variables^1^. Understanding the distribution of functional traits at broad spatial scales can reveal the close relationships between organisms and their biotic and abiotic environments^2^. Many large-scale studies on plant functional traits and distribution patterns have been reported in recent decades. These studies have primarily examined traits that influence plant growth, survival and reproduction, such as leaf area^3^, seed size^4^, plant height^5^ and plant phenology^6^. However, large-scale distribution patterns of fruit type and the important environmental drivers affecting them remain poorly understood. Additionally, it is unclear whether the geographic distribution of fleshy-fruited species and the environmental determinants differ among various growth forms (i.e., herbaceous species vs. woody species).

Fruit type is a key ecological trait that influences many aspects of a species’ reproductive strategy, including pre-dispersal predation^7,8^, seed dispersal syndrome^9^ and seed dispersal distance^10,11^. Differences in seed dispersal indirectly influence plant populations and community dynamics because they determine the initial template of spatial distribution^12^. For example, plants species dispersed by animals gain an advantage of longer distance dispersal^11^ than those dispersed by abiotic conditions like wind^13^. Ecological and evolutionary research focusing on fleshy-fruited species is relatively extensive^14–17^, however, the majority of research has focused on interactions between fleshy fruits and frugivores^8,9,18^ and variation in fleshy-fruit characteristics^2,7,19,20^. Few quantitative studies focus on the spatial distribution of fleshy-fruited species at larger scales, and fewer take growth form into account.

Climate plays a central role in the distribution of plant species^21^. For fleshy-fruited species, climate is especially important because fleshy fruit evolution is driven by vegetation changes caused by climatic variation^22^. Many studies investigating the relationship between fleshy-fruited species and abiotic factors at a local scale have found that precipitation and temperature are the two most important environmental factors for the growth of fleshy-fruited plants. Fleshy-fruited species generally produce fruits in the wet season^19^ and the proportion of species with fleshy fruits is higher in wet, warm areas^14,23,24^. With respect to climate variation, fleshy-fruited species tend to thrive in places where the climate is stable. Since fleshy fruits contain high levels of water and organic compounds^25^, large temperature fluctuations, especially cold temperatures, can result in damage to fruits that affects reproductive success^26^. However, it remains unclear which climate factors contribute the most to fleshy-fruited species distribution.

Different plant functional types play different roles in matter and energy processes and overall ecosystem functioning^27^. To advance our understanding of species richness patterns along environmental gradients we also need to compare patterns by growth form^28^. For fleshy-fruited species, species from different growth forms may also have different relationships with environmental factors^29^. Although fruit traits may be similar, other plant characteristics may have a greater influence on the relationship between fruit type and the environment. For example, woody species have bigger fruit size and taller heights, and as a result may need more water to construct pulp and transport nutrients. Herbaceous species may acquire energy more rapidly to accumulate biomass for organ growth due to their limited lifetime^30^ compared with the woody species. However, at this time the influence of growth form on the relationship between fruit type and climatic factors is poorly studied.

China covers a large geographic area with a great diversity of climates and steep environmental gradients. The precipitation and temperature gradients exist from wet and warm in the southeast, to cold and dry in the northwest. A topographic gradient follows from high plateaus in the west to flatlands in the east. These climatic and geographic characteristics allow us to assess distribution patterns of fleshy-fruited species across large environmental gradients. We hypothesize that climate is the key driver of fleshy-fruited species distribution and that the greater proportion of fleshy-fruited species will follow climate gradients with significant latitude, longitude and altitude patterns in China. Water surplus is proposed as a prerequisite condition for fleshy-fruited species evolution^31^, and warm temperatures provide energy for the development of fleshy-fruited species^32,33^. Thus, we hypothesize that the proportion of fleshy-fruited species is higher in southeast China, in areas at lower altitudes and with abundant rainfall and mild temperatures.

As for the influence of growth form on the relationship between fruit type and climate, we hypothesize that the most significant climatic determinants differ between woody and herbaceous fleshy-fruited species. Primarily, woody and herbaceous species are characterized by different adaptations to water limitation^34^. For example, woody plant growth has been shown to be more sensitive to spatial variation in rainfall than for herbaceous plants^35^. However, in rainy areas, other environmental factors may gain relative importance in determining species distribution. Herbaceous plants generally dominate at higher latitudes, are more correlated to energy variables^36^, and have lower precipitation requirements. Thus, for herbaceous fleshy-fruited species, temperature may be the most important limiting factor relative to precipitation. For example, temperature is the most important factor in determining *Salix* species distribution^37^, as most species occur along rivers where water is not a limiting factor^35^. Our study is the first to assess whether the same environmental variables drive both woody and herbaceous fleshy-fruited species distribution.

In this study, we examine the proportions of plant species bearing fleshy fruits for both woody and herbaceous species and assess how the proportions of fleshy-fruited species vary along spatial and climatic gradients across China. We use information on fruit types and spatial distributions for over 27000 species in China. Specifically, we aim to: (1) examine the broad-scale geographical patterns (latitude, longitude, altitude) of fleshy-fruited species in China; (2) evaluate the relative importance of climatic variables to explain these geographical patterns; and (3) assess whether the geographic patterns and their determinants are consistent between different growth forms (woody versus herbaceous species).

## Results

### Spatial patterns

**Figure 1:**
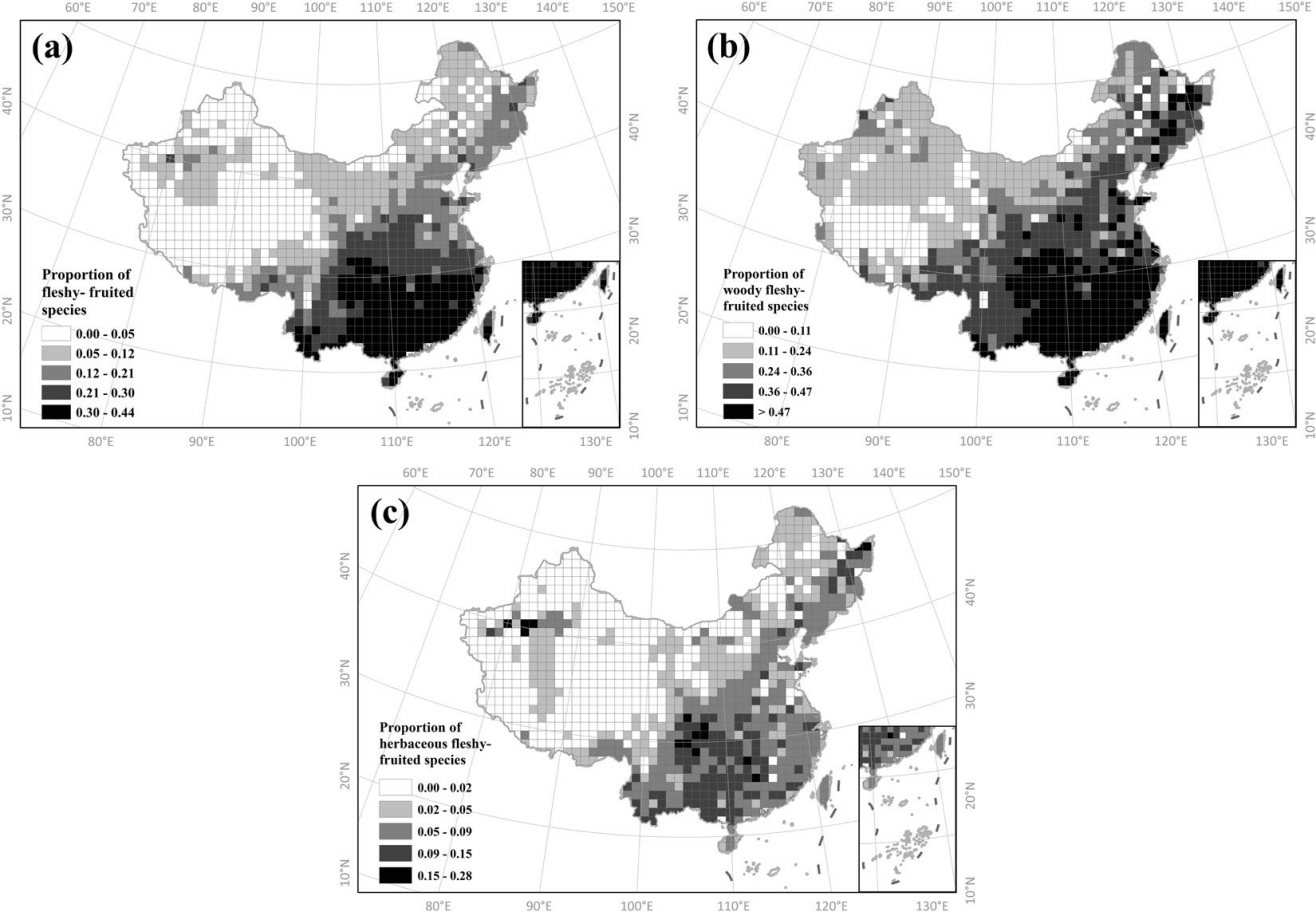
Geographical patterns of the distribution of fleshy-fruited species in China estimated in grids of 100 × 100 km. (**a**) Total species (**b**) Woody species (**c**) Herbaceous species.

The proportion of fleshy-fruited species is higher in southeast China and this pattern is consist for woody and herbaceous species (Fig. 1). The proportion of fleshy-fruited species for all species pooled ranges from 0 to 44.2%. The proportion of fleshy-fruited species was significantly higher for woody species compared to herbaceous species (t = 56.31, P < 0.001, Fig. 1b,c), with the proportion ranging from 0 to 83.3% and 0 to 28.6%, respectively. The proportion of fleshy-fruited species tend to be higher at lower latitudes, greater longitudes and lower altitudes (Fig. S1). Interestingly, the relationship between the proportion of fleshy-fruited species with latitude and longitude is nonlinear, just as with the environmental variables (Figs S2 and S3).

**Figure 2:**
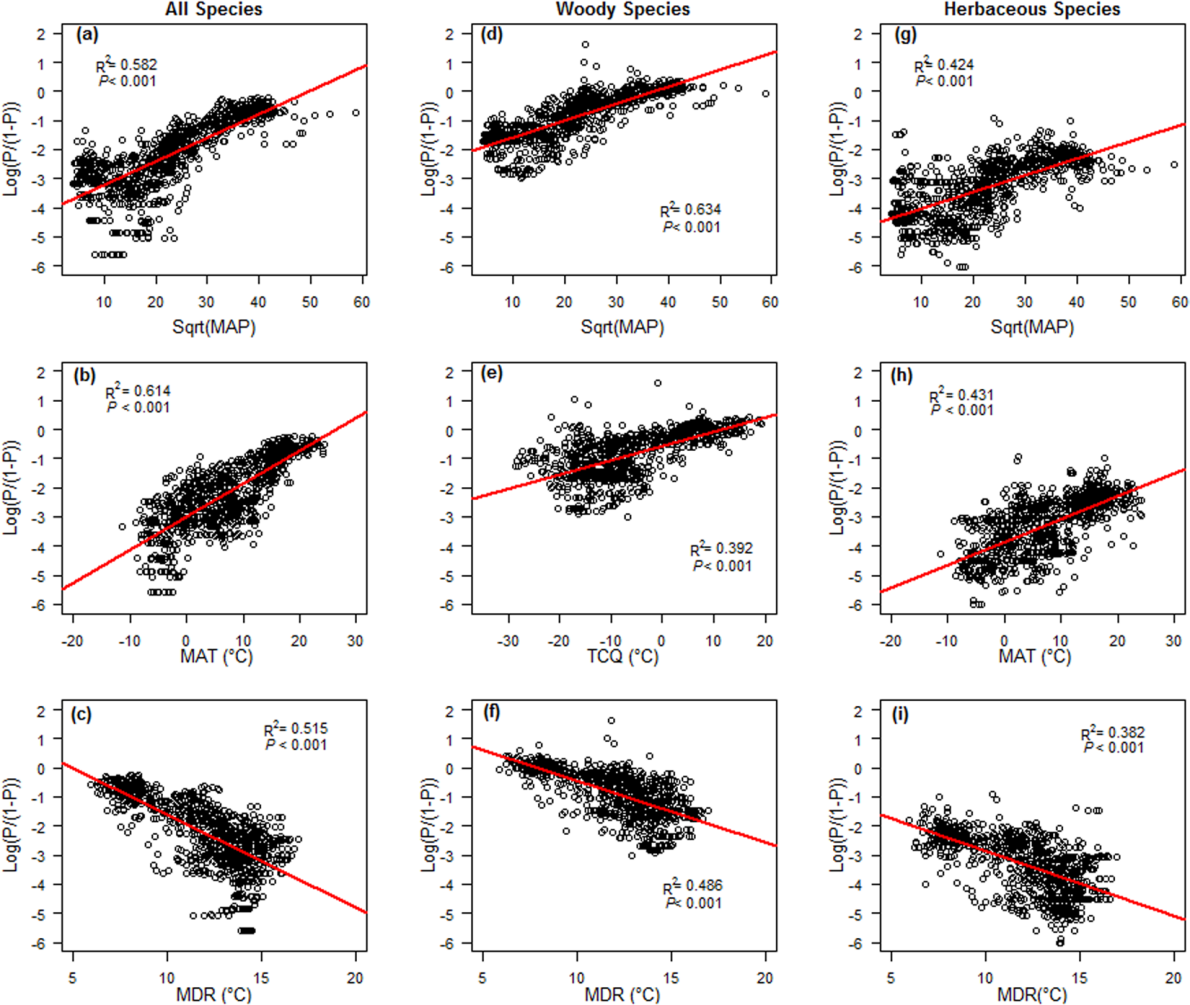
The ordinary least square (OLS) regression between the proportion of all, woody and herbaceous fleshy-fruited species and nine main climate variables. (**a**–**c**) All species, (**d**–**f**) Woody species, (**g**–**i**) Herbaceous species. ‘P’ stands for the proportion of fleshy-fruited species, which were logit-transformed in the regression analysis. Precipitation variables for MAP: mean annual precipitation were square root transformed in the regression analysis. MAT: mean annual temperature; TCQ: temperature of the coldest quarter; MDR: mean diurnal range.

### Environmental variables

Overall, the proportion of fleshy-fruited species was positively correlated with temperature and precipitation variables, but negatively correlated with climatic variability (Fig. 2). Specifically, mean annual temperature (MAT), mean annual precipitation (MAP), and mean diurnal range (MDR) had the strongest correlation with the proportion of fleshy-fruited species among the three categories of the climatic variables (Pseudo-*R*^2^ = 0.614, 0.583 and 0.515 respectively in Table 1; Fig. 2a–c; Table S2). The combined model that included the three most correlated climatic variables in each climate group (MAT, MAP, MDR) and their interactions, accounted for 72.3% of the variation in the proportion of fleshy-fruited species (Table 2). MAT had the greatest significant relative importance for the proportion of fleshy-fruited species, which was consist in simultaneous autoregressive error (SAR) and ordinary least squares (OLS) models (Tables 2, S3). For woody species, MAP, temperature of the coldest quarter (TCQ) and MDR showed the strongest correlation with the proportion of fleshy-fruited species among the three categories of the climatic variables (Pseudo-*R*^2^ = 0.633, 0.392, and 0.486 respectively in Table 1, Fig. 2d–f; Table S2). The combined model that included the three most correlated climatic variables and their interactions, explained 64.1% of the variation in the proportion of fleshy-fruited species. MAP had the greatest significant unique-*R*^2^ for the proportion of woody fleshy-fruited species for the SAR and OLS models (Tables 2, S3). The proportion of herbaceous fleshy-fruited species was correlated most strongly with MAT, MAP and MDR among the three categories of the climatic variables (Pseudo-*R*^2^ = 0.431, 0.424 and 0.382 respectively in Table 1, Fig. 2g–i; Table S2). The combined model that included the three most correlated climatic variables and their interactions, accounted for 49.2% of the variation in the proportion of fleshy-fruited species for the SAR model. MAT had the most significant contribution in affecting the proportion of herbaceous fleshy-fruited species for the SAR and OLS model (Tables 2; S3). Moran’s I were all close to 0 and the P-values of the Moran’s I tests were all greater than 0.1 in the SAR models, indicating that the SAR models successfully removed the spatial autocorrelation from the model residuals of OLS models (Tables 1, 2; Figs S4–S6).

**Table 1:** Results of spatial linear models (SAR model) of environmental variables and the proportion of fleshy-fruited species for all plants pooled, woody species, and herbaceous species. The proportion of fleshy-fruited species was logit-transformed in the analysis. Pseudo-*R*^2^: the squared Pearson correlation between observed and predicted fleshy-fruited species proportions of full models.

	All plants pooled				Woody species				Herbaceous species			
	z	Pseudo-*R*^2^	AIC	Moran’s I	z	Pseudo-*R*^2^	AIC	Moran’ I	z	Pseudo-*R*^2^	AIC	Moran’s I
**Precipitation**												
Sart (MAP)	0.89	58.3	1292	0.006	5.83***	63.3	990	0.002	0.36	42.4	1438	0.007
Sqrt (PWQ)	0.53	52.6	1299	0.009	5.16***	60.7	995	0.001	−0.28	40.3	1442	0.009
Sqrt (PDQ)	1.22	48.7	1293	0.008	4.85***	46.8	996	0.006	1.89	34.2	1437	0.009
**Temperature**												
MAT	5.51***	61.4	1275	0.004	1.06	37.9	1021	0.005	8.06***	43.1	1375	0.002
TCQ	5.83***	55.8	1263	0.001	2.01*	39.2	1015	0.005	8.29***	34.4	1372	0.002
TWQ	4.94***	44.1	1275	0.004	0.01	22.2	1017	0.004	7.62***	36.9	1378	0.003
**Variability**												
MDR	1.18	51.5	1294	0.009	−3.04	48.6	1575	0.005	−0.89	38.2	1435	0.006
PS	−1.70	24.7	1291	0.009	−2.89	20.5	1011	0.004	2.99**	12.1	1429	0.008
TS	−0.06	12.0	1295	0.008	−4.23	14.9	1003	0.002	1.15	4.40	−1428	0.004

*** indicates P < 0.001, **0.001 < P < 0.01, *0.01 < P < 0.05.

All the P-values of the Moran’s I tests for the SAR models were greater than 0.1. Precipitation variables for MAP: mean annual precipitation, PWQ: precipitation of wettest quarter and PDQ: precipitation of driest quarter, were square root transformed in the analysis. MAT: mean annual temperature; TCQ: temperature of the coldest quarter; TWQ: mean temperature of warmest quarter; MDR: mean diurnal range; PS: precipitation seasonality; TS: temperature seasonality. Dev: percentage deviance explained by the models.

**Table 2:** Results of spatial multivariable linear models (SAR model) of environmental variables and the proportion of fleshy-fruited species for all plants, woody species and herbaceous species.

	Z	Unique-*R*^2^	Pseudo-*R*^2^	AIC	Moran’s I
**All plants**					
Sqrt(MAP)	1.66	0.106	—	—	—
MAT	5.49***	0.687	—	—	—
MDR	0.52	0.698	—	—	—
	—	—	72.3	1354	0.006
**Woody species**					
Sqrt(MAP)	5.08***	0.119	—	—	—
TCQ	2.60**	0.000	—	—	—
MDR	−1.44	0.000	—	—	—
	—	—	64.1	986	0.001
**Herb plants**					
Sqrt(MAP)	0.59	0.014	—	—	—
MAT	8.57***	0.080	—	—	—
MDR	−1.99*	0.091	—	—	—
	—	—	49.2	1375	−0.002

*** indicates P < 0.001, **0.001 < P < 0.01, *0.01 < P < 0.05.

The proportion of fleshy-fruited species was logit-transformed in the analysis. All the P-values of the Moran’s I tests for the SAR models were greater than 0.1. MAP: mean annual precipitation was square root transformed in the analysis. MAT: mean annual temperature; TCQ: temperature of the coldest quarter; MDR: mean diurnal range. Pseudo-*R*^2^: the squared Pearson correlation between observed and predicted fleshy-fruited species proportions of full models. Unique-*R*^2^: differences between the *R*^2^ from full SAR models and that from SAR models without that predictor. ‘—’: no value.

## Discussion

Overall, the geographic patterns of fleshy-fruited species were generally similar between growth forms with differences explained by links to different environmental drivers. MAP had the greatest influence on woody fleshy-fruited species distribution and MAT was most important for the herbaceous species distribution.

The proportion of fleshy-fruited species tend to be higher in the southeast zones in China, where climate is moist, warm and mild. Interestingly, the latitude and longitude gradients were nonlinear. The significant latitudinal gradient was only observed in the tropics and subtropics (18.8°N-35°N) (Fig. S1), and the proportion decreased with increasing latitude. These patterns are similar to recent findings from the southern hemisphere in Australia, which also has a broad latitudinal range (9.2°S–43.7°S) including both tropical and temperate biomes^38^. It is possible that the latitudinal pattern could have existed in tropical and subtropical zones because a simple linear regression was used and accurate latitudinal trends may have been masked by the overall trend of the tropical to temperate zone transition. Another study conducted near the equator failed to find a significant latitudinal trend in the proportion of fleshy-fruited species across 13° of latitude (12.5°S–25.5°S) in tropical forests^39^, which likely resulted from the relative homogeneity of the climatic zone.

Admittedly, other factors besides climate factors may explain these geographic patterns. For example, historical factors controlling patterns such as recolonization after deglaciation may also have had strong effects on the current distribution patterns. We expect recolonization to be faster for herbaceous than woody species, which could explain why there is a strong shift in the proportion of fleshy-fruited woody species between temperate and tropical zones. Due to fast recolonization by herbaceous species, their latitudinal relationship is weaker than woody species, which could also be strongly linked to glacial refugia^40^. Altitudinal patterns of fleshy fruit species may be influenced by the distribution of vertebrate species on which they rely for seed dispersal^15^. For example, the presence of mastozoochory tends to decrease with increasing altitude^39^. All in all, these geography patterns were consistent with the climatic distribution patterns (Figs S2 and S3). This confirmed our hypothesis that climatic factors were very important in shaping the fleshy-fruited species distributions.

The geographic patterns were consistent between woody and herbaceous species. This indicates that growth form has little effect on the distribution of fleshy-fruited species. However, the proportion of woody fleshy-fruited species was greater than herbaceous species. This may be due to the difference in reproduction strategy between woody and herbaceous species. For smaller, herbaceous plants, the relative resource cost of producing fleshy fruits is higher than for woody species^22^. The smaller size of herbaceous plants results in smaller seeds^41,42^, which disperse more easily even if unassisted^22^. The higher dispersion of the smaller seeds reduces the payoff of fleshiness for smaller plants. In contrast, woody angiosperms are often recruited to habitats that favor larger seeds requiring assistance for sufficient dispersal, so the fleshiness of the fruit is increased^22^.

Our findings are consistent with previous studies conducted at both local^14,24^ and regional scales^38,39^. The proportion of fleshy-fruited species is higher in wet, warm and stable environments. This supports the hypothesis that abiotic factors play an important role in shaping fruit type^23^ and determining the distribution of fleshy-fruited species^38^. What makes our study unique is that we considered the different growth forms when comparing the relationship between fleshy-fruited species and climatic factors. Thus, we could also identify the main climatic factors and their relative contribution in driving the distribution of fleshy-fruited species.

Growth form influences the relationship between fleshy-fruited species and climatic factors. The model results showed that mean annual temperature is the most important climatic variable in shaping the distribution of fleshy-fruited species for all species and herbaceous species, while mean annual precipitation is the most important for woody fleshy-fruited species. This result is consistent with previous research that found that precipitation has a strong effect on woody species richness, but temperature is more impactful for herbaceous species^29^. Woody species prefer tropical, subtropical and warm temperate zones, where water availability generally controls species distribution^36^. Meanwhile, herbaceous plants primarily dominate higher latitudes and drier climate zones, where both water and energy availability drive species distribution^36^. Márquez, Real & Vargas^43^ studied the broad-scale geographical variation in fleshy-fruited plant species richness in Europe, and found that energy, rather than precipitation, was the main climatic driver for fleshy-fruited species richness. However, this study focused mainly on herbaceous species and did not consider the influence of growth form.

There are two possible explanations for why the chosen climatic variables have differing importance on the distribution of woody and herbaceous fleshy-fruited species. Firstly, woody plants and herbs differ in many characteristics that influence their relationship with the environment. For example, woody plants have bark to withstand cold temperatures, but they need adequate moisture to maintain nutrient transport and the development of succulent fruit because of their height. In contrast, herbaceous plants are smaller, shorter and able to adapt to drier places than woody plants. However, herbs require more energy over shorter periods of time to accommodate their accelerated metabolic rate, to promote growth and to fulfill their life cycle^30^. Fruit size is another consideration. Herbaceous species require less moisture to construct pulp, so their fruit size is generally smaller than woody species due to an overall smaller plant size^44^. Secondly, according to the Cannikin law, mean annual precipitation may be the limiting factor for woody fleshy-fruited species distribution in our study area in China, but mean annual temperature appears to be the limiting factor for herbaceous fleshy-fruited species, as indicated by their concentrated distribution in southeast China where water is plentiful.

The proportion of fleshy-fruited species was negatively related to MDR, temperature seasonality (TS) and precipitation seasonality (PS), regardless of plant growth form. This indicates that stable climates favor fleshy-fruited species growth which is consistent with results from Australia^35^. Mean diurnal range was the most important factor explaining fruit type variation. The high moisture content in fleshy fruits means that large diurnal temperature fluctuations, especially during the fruiting season, can cause physical damage to the pulp, which impacts reproductive success.

We use the largest compiled datasets to date of species distribution data in China to explore the environmental determinants of fleshy-fruited species distribution, comparing the two different growth forms. Our results support the water-energy dynamic hypothesis suggesting that species diversity is correlated with water availability and temperature^45^. Water and energy are essential for plant growth and reproduction^46^. Improving our understanding of the climatic variables most important in shaping ecological patterns is critical to accurately predicting the effects of climate change on these ecosystems^38^. Our findings may enhance predictions for future fleshy-fruited species distributions. With future global climate change, precipitation and temperature regimes are expected to increase^47^ and mean diurnal range are expected to decrease^48^. If these circumstances prove true, the fleshy-fruited species could increase in range in areas where temperature and precipitation increase and climatic variation decreases in the future. Further, species with different growth forms will have different responses to climate change: woody fleshy-fruited species may be more sensitive to precipitation change than to temperature change, as herbaceous species are.

Although fleshy-fruited species distributions rely on abiotic factors, certain biological mechanisms that were not considered in this study may also play a role in their existing distribution patterns. Firstly, warm and wet environments favor forest cover, which selects for shade tolerance, large seeds and animal disperal^49,50^. Thus, a fleshy-fruited species that is dispersed by animals would be more likely to grow in mild regions with their dispersers. Secondly, fleshy-fruited species distribution could also be related to frugivore diversity. For example, Lavabre *et al*.^51^ found that the functional diversity of frugivorous birds may shape the spatial pattern of seed dispersal for a relic plant species. Márquez, Real & Vargas^43^ found that frugivore richness has a significant influence on the local presence of fruiting plant species. However, because frugivore data is not publicly available for China and the large database containing more than 27000 plant species is so spatially broad, it is impossible to account for all these abiotic and biotic factors in a single study. Here we hope to provide a basis for further research regarding complex animal-vegetation-climate interactions. Identifying the relative influence of biotic and abiotic processes affecting the distribution patterns of fleshy-fruited species presents an interesting challenge for further research efforts.

In summary, we assessed the biogeographic patterns of fleshy-fruited species and the underlying environmental drivers among different growth forms in tropical and temperate zones of China. Our results show that the proportion of fleshy-fruited species follow significant latitudinal, longitudinal, and altitudinal patterns, which are consistent for both woody and herbaceous species. Results also suggest that these patterns are driven by large scale environmental gradients related to temperature and precipitation variables. Mean annual precipitation had the greatest contribution to woody-fruited species distribution, while mean annual temperature was identified as the greatest contributor for herbaceous species. This work advances our understanding of the relationships between fleshy-fruited species and environmental gradients for different growth forms and provides a basis and reference for the influence of climate change on fleshy-fruited species distributions and fruit-frugivore interactions.

## Methods

### Species distribution data

Species distribution data was extracted from the Chinese Vascular Plant Distribution Database, which assembled species distribution information of over six million specimens and over 1000 volumes of published books, such as *Flora of China* and regional plant resource investigation reports^52^. The database provides distribution information for over 30000 species across all counties in China. Plant names were standardized with *Catalogue of Life China* (Checklist 2015, http://www.sp2000.org.cn/) and *Flora of China* (http://foc.eflora.cn/). Cultivated and alien species were excluded from the dataset. We categorized the fruit of each species as either fleshy or non-fleshy, according to species descriptions in *Flora Reipublicae Popularis Sinicae* (http://frps.eflora.cn/) and *Flora of China*.

### Fruit type data

For each species we classified fruit type into one of sixteen categories: utricle, samara, schizocarp, citrus fruit, follicles, stone fruit, gourd fruit, pods, nuts, berries, pods, pome, achene, cremocarp, capsule, or caryopsis. Citrus fruit, stone fruit, gourd fruit, berries, and pome were classified as fleshy fruits. The others were classified as dry fruits. In total there were 27941 angiosperm species (2580 Genus, 224 Family) that had both fruit type and distribution information, with 5435 fleshy fruit species.

Growth forms were classified as woody or herbaceous species according to species descriptions in *Flora Reipublicae Popularis Sinicae* and *Flora of China*. There were 27803 (11222 woody species, 16581herbaceous species) species that had growth form, fruit type and distribution information. We included 4692 woody fleshy-fruited species and 727 herbaceous fleshy-fruited species.

### Climatic data

We examined the influence of nine climate variables on the distribution of fleshy-fruited species and grouped them into three categories: (i) Temperature variables, including mean annual temperature (MAT), temperature of the coldest quarter (TCQ) and temperature of the warmest quarter (TWQ); (ii) Precipitation variables, including mean annual precipitation (MAP), precipitation of wettest quarter (PWQ) and mean precipitation of the driest quarter (PDQ); (iii) Climatic variability, including temperature seasonality (TS; standard deviation), precipitation seasonality (PS; coefficient of variation) and mean diurnal range MDR; (monthly mean (max temperature − min temperature)). Temperature, precipitation and altitudinal data were extracted from the WorldClim database at a spatial resolution of 30 arc-seconds^53^.

### Statistical analyses

To eliminate the influence of area and administrative boundaries, we projected species distribution data to the Albers equal-area grid system with a resolution of 100 km × 100 km. We only used grid cells with an area >3000 km^2^ to avoid sampling bias. In total 984 grid cells were used in this study. We then calculated the proportion of fleshy-fruited species to total species for each grid cell. Similarly, we calculated the mean value of climatic variables for each grid cell. Because the independent variable was proportional, we logit-transformed the proportions of fleshy-fruited species. All precipitation variables were square-root-transformed to improve linearity and normality of model residuals.

To examine spatial patterns of fleshy-fruited species, we analyzed the relationship between the proportion of fleshy-fruited species and latitude, longitude and altitude individually using simple linear regression and piecewise regression. The piecewise regression models for latitude and longitude fit better than simple linear regression analysis (Table S1), thus we only present the piecewise regression model results (Fig. S1). Piecewise regression analyses were run in R v3.4.1^54^ using the package SEGMENTED^55^. Similarly, we did a piecewise regression analysis between latitude, longitude and the climate variables (Figs S2 and S3).

The relationship between the proportion of all, woody, and herbaceous fleshy-fruited species, and each climate variable were analyzed separately using ordinary least squares (OLS). To identify which of the environmental variables influenced the proportion of the fleshy-fruited species the most, we selected the best single predictor variable which had the greatest *R*^2^ from each category and constructed a multivariate model. Variance inflation factors (VIFs) were also calculated for all models to evaluate the significance of multi-collinearity^56^ for the OLS model, using the ‘vif’ function in package CAR in R^57^.

Due to the strong spatial autocorrelation of residuals of OLS (Tables S2, S3; Figs S4–S6), we performed spatial linear regression. We used Moran’s I statistic to determine if spatial autocorrelation was present in the residuals of the OLS and spatial linear models. We used the simultaneous autoregressive error (SAR) model to account for the spatial autocorrelation structure in model residuals^58^. The SAR model is considered one of the best spatial models available^59^ and has been used in numerous studies^60^. We performed SAR models with different neighborhood structures and spatial weights (lag distances between 200 and 1,500 km neighborhood). Final model selection was based on the reduction of spatial autocorrelation in residuals and a minimization of AIC values. SAR of the spatial error model type with a lag distance of 300 km and weighted neighborhood structure was the best spatial structure for all species. A lag distance of 400 km was best for the woody and herbaceous species. The expected Moran’s I value for low spatial autocorrelation is close to 0^61^. Spatial Moran’s I correlograms for the OLS and SAR residuals of all plants pooled, woody species and herbaceous species showed that spatial autocorrelation had been removed (Figs S4–S6).

In addition, we calculated the total and unique contributions of each variable to explaining the variation of the proportion of fleshy-fruited species. We excluded the spatial signal of SAR prediction since our focus was the effects of explanatory variables rather than their joint effects with space. The total contribution was the pseudo-*R*^2^ calculated by the squared Pearson correlation between observed values and the non-spatial trends of fitting by the single predictor SAR model^62^. The unique contribution of each variable was calculated by subtracting the pseudo-*R*^2^ of the SAR model (*R*^2^ for the OLS model) excluding that variable from the pseudo-*R*^2^ of the full SAR model (*R*^2^ for the OLS full model)^52^. The OLS univariate model and multivariable model results were provided in Tables S2, S3. Spatial statistics were performed with the package SPDEP in R^63,64^.

## Electronic supplementary material

Supplementary Information

## Data Availability

The datasets analysed during the current study are not publicly available because the authors do not have the right to open the data, but they are available from the corresponding author on reasonable request.
