# Supplementary material for: Contributions of precipitation and temperature to the large scale geographic distribution of fleshy-fruited plant species: Growth form matters
Source: Sci Rep. 2018 Nov 19;8:17017. doi: 10.1038/s41598-018-35436-x (PMC6243012; doi:10.1038/s41598-018-35436-x)
Supplement: Supplementary file 1 — Supplementary Information [file 41598_2018_35436_MOESM1_ESM.docx]

**Contributions of precipitation and temperature to the large scale geographic distribution of fleshy-fruited plant species: Growth form matters.**

Yuan Zhao^1,3,4^, Honglin Cao^1^, Wubing Xu^3^, Guoke Chen^3^, Juyu Lian^1^, Yanjun Du^2,3*^ & Keping Ma^3^

**Supplementary Information**

**The comparison between the latitude or longitude of the linear and piecewise regression models is depicted in Table S1. The distributions of fleshy-fruited species and the comparison of various climatic factors to latitude and longitude are shown in Figures S1-S3.**

| **Growth**  **Forms** | | **Variables** | | **Linear model** | | | | | **Piecewise Regression Model** | | | | | **ANOVA** | | |
| --- | --- | --- | --- | --- | --- | --- | --- | --- | --- | --- | --- | --- | --- | --- | --- | --- |
|  |  |  |  | *t* | *R^2^* | | AIC | | *t* | *R^2^* | | AIC | | | *P* |  |
| All plants | Latitude | | -16.85*** | | 0.229 | 2764 | |  | -15.92*** | | 0.37 | | 2578 | | <0.001 | |
|  | longitude | | 19.29*** | | 0.28 | 2697 | |  | 21.11*** | | 0.41 | | 2508 | | <0.001 | |
| Woody species | Latitude | | -16.32*** | | 0.225 | 1957 | |  | -16.48*** | | 0.32 | | 1841 | | <0.001 | |
|  | longitude | | 20.46*** | | 0.313 | 1846 | |  | 18.76*** | | 0.40 | | 1731 | | <0.001 | |
| Herbaceous species | Latitude | | -10.97*** | | 0.118 | 2394 | |  | -12.08*** | | 0.22 | | 2282 | | <0.001 | |
|  | longitude | | 19.79*** | | 0.302 | 2182 | |  | 16.03*** | | 0.34 | | 2127 | | <0.001 | |

**Table S1.** The comparison between the latitude or longitude of the linear and piecewise regression models for all plants pooled, woody species and herbaceous species. *** indicates P<0.001, ** 0.001<P<0.01, * 0.01 < P < 0.05


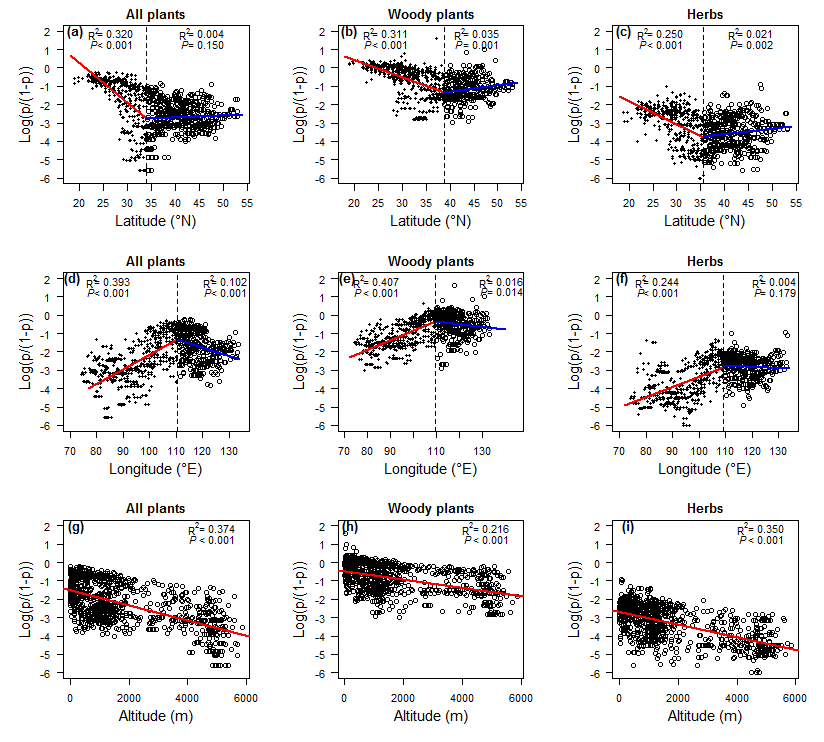


**Figure S1.** Geographic patterns of fleshy-fruited species distribution for all species, woody species, and herbaceous species. Latitudinal patterns: (a) to (c); longitudinal patterns: (d) to (f); altitudinal patterns: (g) to (i). The breakpoints were confirmed by segmented regression for the latitudinal and longitudinal pattern analysis. ‘p’ stands for the proportion of fleshy-fruited species, which were logit-transformed in the regression analysis.

**
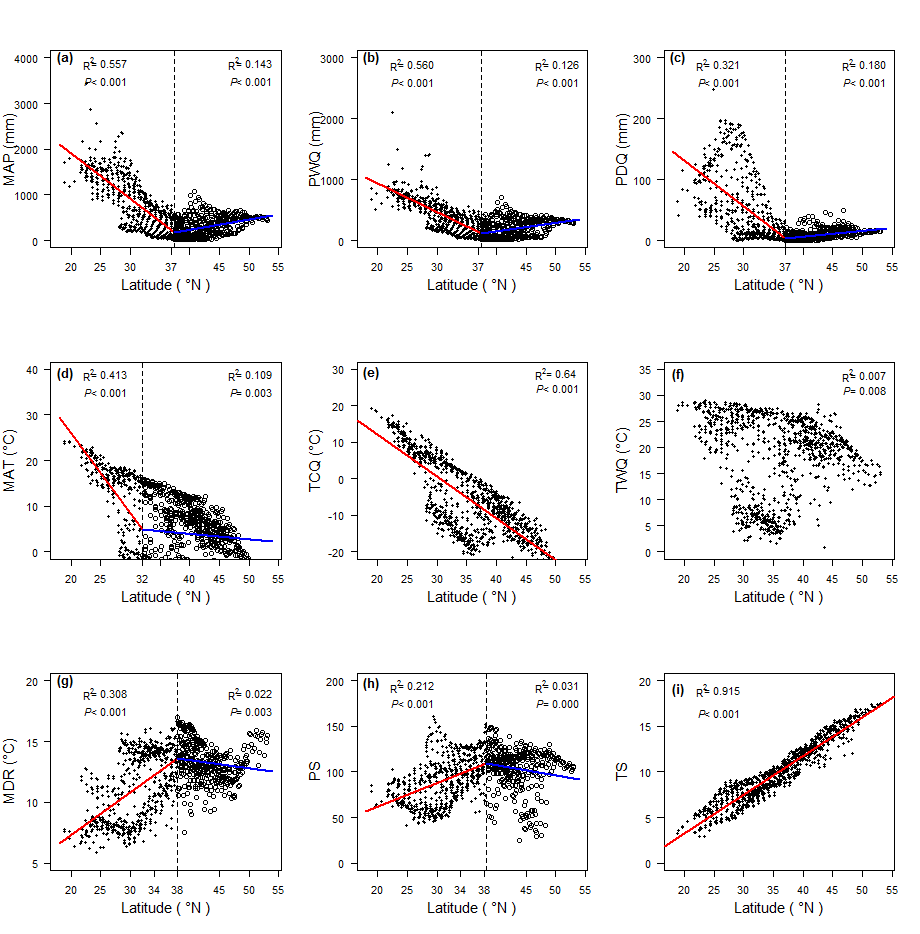
**

**Figure S2.** The regression between latitude and climatic factors in China. MAP: mean annual precipitation; PWQ: precipitation of wettest quarter; PDQ: precipitation of driest quarter; MAT: mean annual temperature; TCQ: temperature of the coldest quarter; TWQ: mean temperature of warmest quarter; MDR: mean diurnal range; PS: precipitation seasonality; TS: temperature seasonality. The breakpoints were confirmed by segmented regression.


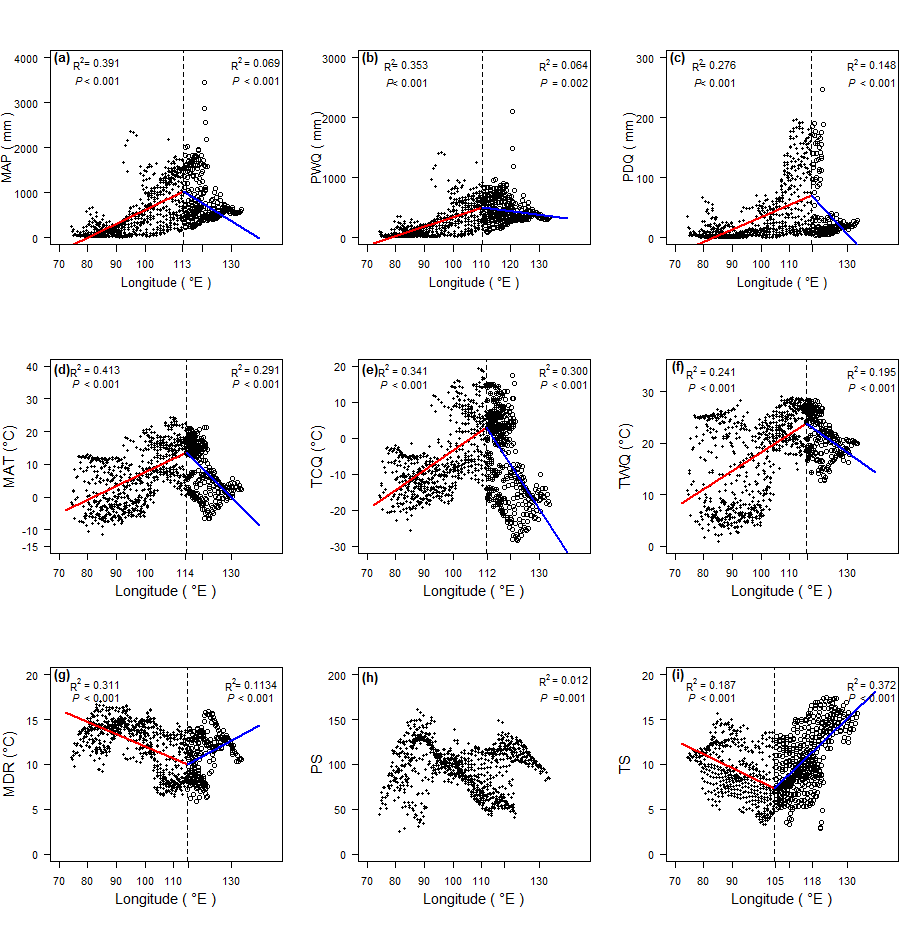


**Figure S3.** The regression between longitude and climatic factors in China. MAP: mean annual precipitation; PWQ: precipitation of wettest quarter; PDQ: precipitation of driest quarter; MAT: mean annual temperature; TCQ: temperature of the coldest quarter; TWQ: mean temperature of warmest quarter; MDR: mean diurnal range; PS: precipitation seasonality; TS: temperature seasonality. The breakpoints were confirmed by segmented regression.

**Least squares linear regression model (OLS) results are depicted in Tables S2-S3.**

|  | | **All plants pooled** | | | | |  | **Woody species** | | | |  | | **Herbaceous species** | | | | | |
| --- | --- | --- | --- | --- | --- | --- | --- | --- | --- | --- | --- | --- | --- | --- | --- | --- | --- | --- | --- |
|  | *t* | | | Dev,% | AIC | Moran's I |  | *t* | Dev,% | AIC | Moran's I | |  | | *t* | Dev,% | AIC | Moran's I |  |
| **Precipitation** |  | | | |  |  |  |  |  |  |  | |  | |  |  |  |  |  |
| Sqrt(MAP) | | | 36.6*** | 58.2 | 2174 | 0.51*** |  | 39.8*** | 63.4 | 1268 | 0.23*** | |  | | 25.7*** | 42.4 | 2009 | 0.39*** |  |
| Sqrt(PWQ) | | | 32.6*** | 52.6 | 2296 | 0.55*** |  | 33.7*** | 60.7 | 1332 | 0.25*** | |  | | 24.7*** | 40.3 | 2041 | 0.40*** |  |
| Sqrt(PDQ) | | | 30.1*** | 48.7 | 2373 | 0.61*** |  | 28.4*** | 46.8 | 1610 | 0.46*** | |  | | 21.7*** | 34.3 | 1432 | 0.48*** |  |
| **Temperature** | | |  |  |  |  |  |  |  |  |  | |  | |  |  |  |  |  |
| MAT | | | 39.1*** | 61.4 | 2098 | 0.46*** |  | 23.7*** | 37.9 | 1754 | 0.47*** | |  | | 26.2*** | 43.1 | 1997 | 0.46*** |  |
| TCQ | | | 34.7*** | 55.8 | 2230 | 0.60*** |  | 24.3*** | 39.2 | 1734 | 0.52*** | |  | | 21.8*** | 34.4 | 2126 | 0.58*** |  |
| TWQ | | | 27.5*** | 44.2 | 2454 | 0.62*** |  | 16.2*** | 22.2 | 1961 | 0.57*** | |  | | 19.7*** | 28.5 | 2091 | 0.49*** |  |
| **Variability** | | |  |  |  |  |  |  |  |  |  | |  | |  |  |  |  |  |
| MDR | | | -31.9*** | 51.5 | 2318 | 0.51*** |  | -29.6*** | 48.6 | 1014 | 0.38*** | |  | | -23.6*** | 38.2 | 2073 | 0.41** |  |
| PS | | | -17.7*** | 24.7 | 2740 | 0.72*** |  | -15.4*** | 20.5 | 1981 | 0.62*** | |  | | -11.2*** | 12.1 | 2391 | 0.61*** |  |
| TS | | | -11.4*** | 12 | 2890 | 0.80*** |  | -12.7*** | 14.9 | 2044 | 0.68*** | |  | | -6.4*** | 4.3 | 2467 | 0.66*** |  |

**Table S2.** Results from linear regression models (OLS) of environmental variables and the proportion of fleshy-fruited species for all plants pooled, woody species and herbaceous species. The proportion of fleshy-fruited species was logit-transformed in the regression analysis. Precipitation variables for MAP: mean annual precipitation, PWQ: precipitation of wettest quarter and PDQ: precipitation of driest quarter, were square root transformed in the analysis. MAT: mean annual temperature; TCQ: temperature of the coldest quarter; TWQ: mean temperature of warmest quarter; MDR: mean diurnal range; PS: precipitation seasonality; TS: temperature seasonality. Dev: percentage deviance explained by the models. ***indicates P<0.001, ** 0.001<P<0.01, * 0.01 < P < 0.05

|  | ***t*** | **VIF** | **Unique-*R^2^*** | **Dev,%** | **AIC** | **Moran's I** |
| --- | --- | --- | --- | --- | --- | --- |
| **All plants** | | | | | | |
| Sqrt(MAP) | 15.5*** | 3.08 | 0.06 | - | - | - |
| MAT | 22.4*** | 1.87 | 0.13 | - | - | - |
| MDR | -0.5 | 3.56 | 0 | - | - | - |
|  | - | - | - | 74.4 | 1711 | 0.316*** |
| **Woody species** | | | | | | |
| Sqrt(MAP) | 17.71*** | 3.28 | 0.121 | - | - | - |
| TCQ | 4.15*** | 2.12 | 0.006 | - | - | - |
| MDR | -2.9** | 3.29 | 0.003 | - | - | - |
|  | - | - | - | 66.32 | 1236 | 0.32*** |
| **Herbaceous species** | | | | | | |
| Sqrt(MAP) | 9.1*** | 3.10 | 0.04 | - | - | - |
| MAT | 12.6*** | 1.90 | 0.08 | - | - | - |
| MDR | -1.2 | 3.55 | 0.00 | - | - | - |
|  | - | - | - | 52.8 | 1832 | 0.379*** |

**Table S3.** Results of multiple linear regression (OLS) models of environmental variables and the proportion of fleshy-fruited species for all plants, woody species and herbaceous species. The proportion of fleshy-fruited species was Log transformed in the regression analysis. MAP: mean annual precipitation, was square root transformed in the analysis; MAT: mean annual temperature; TCQ: temperature of the coldest quarter; MDR: mean diurnal range. Unique-*R^2^*: differences between the *R^2^* from full OLS models and that from OLS models without that predictor. VIF: variance inflation factor, evaluate the significance of multi- collinearity. If VIF is greater than five, the multi-collinearity is considered significant. Dev: percentage deviance explained by the models. ‘ - ’: no value. *** indicates P<0.001, ** 0.001<P<0.01, * 0.01 < P < 0.05.

**Moran's I correlograms for OLS and SAR models (Figure S4-S6).**


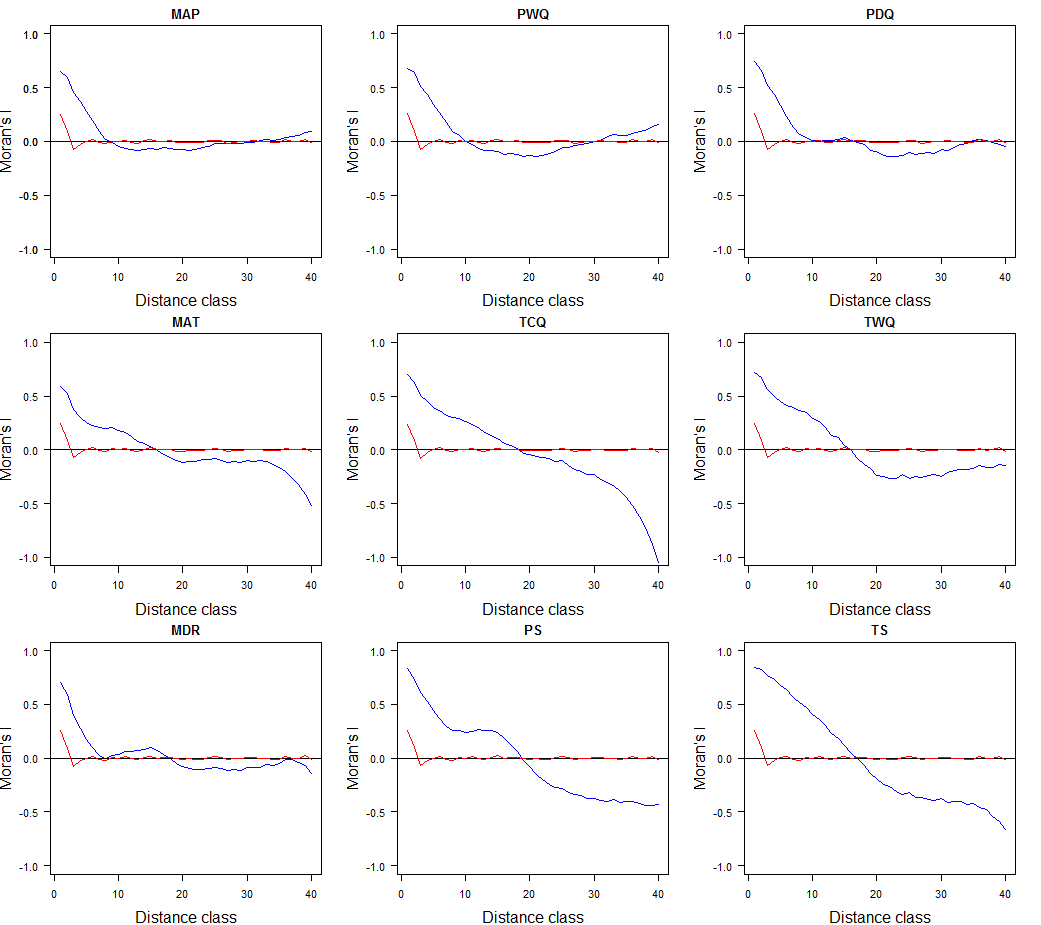


**Figure S4.** Moran's I correlograms for residuals of ordinary least squares regression models (blue) and residuals of spatial autoregressive error models (red) for all the species counted. Both models included the proportion of fleshy-fruited species for all plants and the environment variables for MAP: mean annual precipitation; PWQ: precipitation of wettest quarter; PDQ: precipitation of driest quarter; MAT: mean annual temperature; TCQ: temperature of the coldest quarter; TWQ: mean temperature of warmest quarter; MDR: mean diurnal range; PS: precipitation seasonality; TS: temperature seasonality, respectively. The proportion of fleshy-fruited species was Log transformed in the analysis. All the precipitation variables were square root transformed before doing the analysis. One unit distance class corresponds to 100 km.


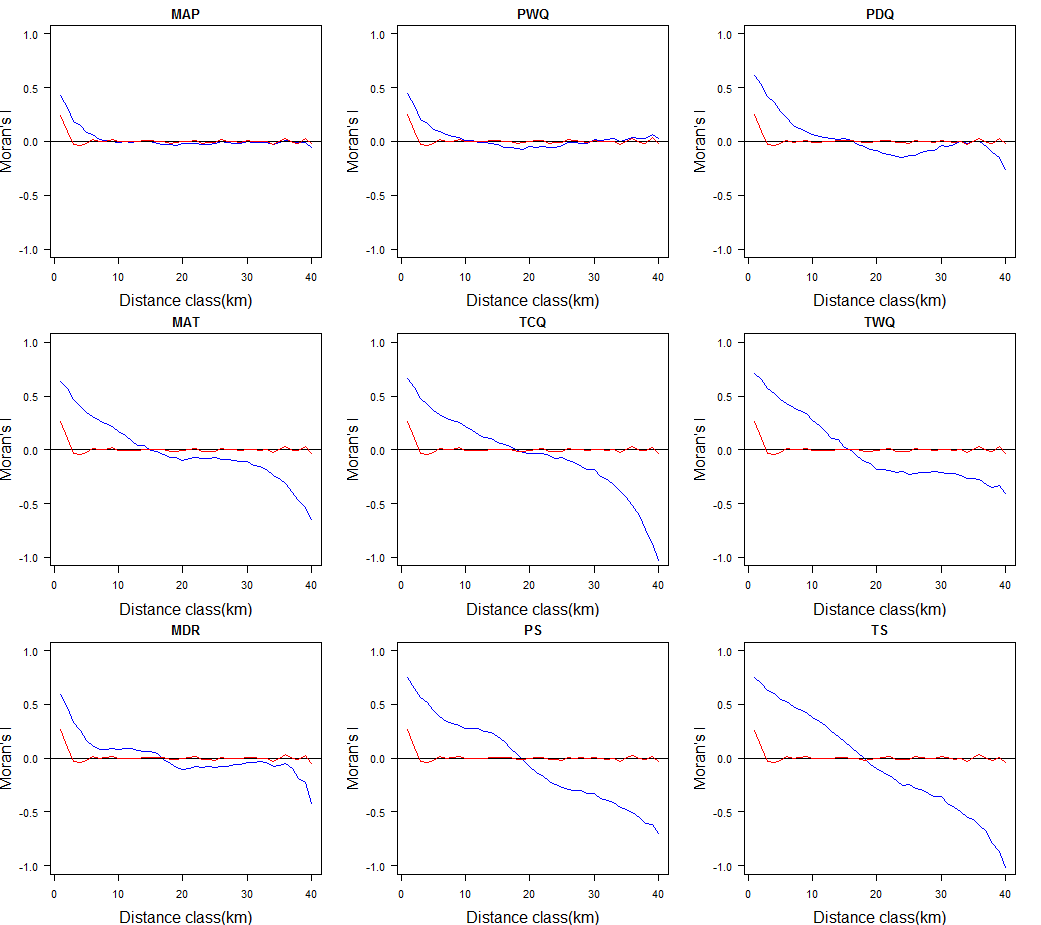


**Figure S5.** Moran's I correlograms for residuals of ordinary least squares regression models (blue) and residuals of spatial autoregressive error models (red) for the woody species. Both models included the proportion of fleshy-fruited species for woody species and the environment variables for MAP: mean annual precipitation; PWQ: precipitation of wettest quarter; PDQ: precipitation of driest quarter; MAT: mean annual temperature; TCQ: temperature of the coldest quarter; TWQ: mean temperature of warmest quarter; MDR: mean diurnal range; PS: precipitation seasonality; TS: temperature seasonality, respectively. The proportion of fleshy-fruited species was Log transformed in the analysis. All the precipitation variables were square root transformed before doing the analysis. One unit distance class corresponds to 100 km.


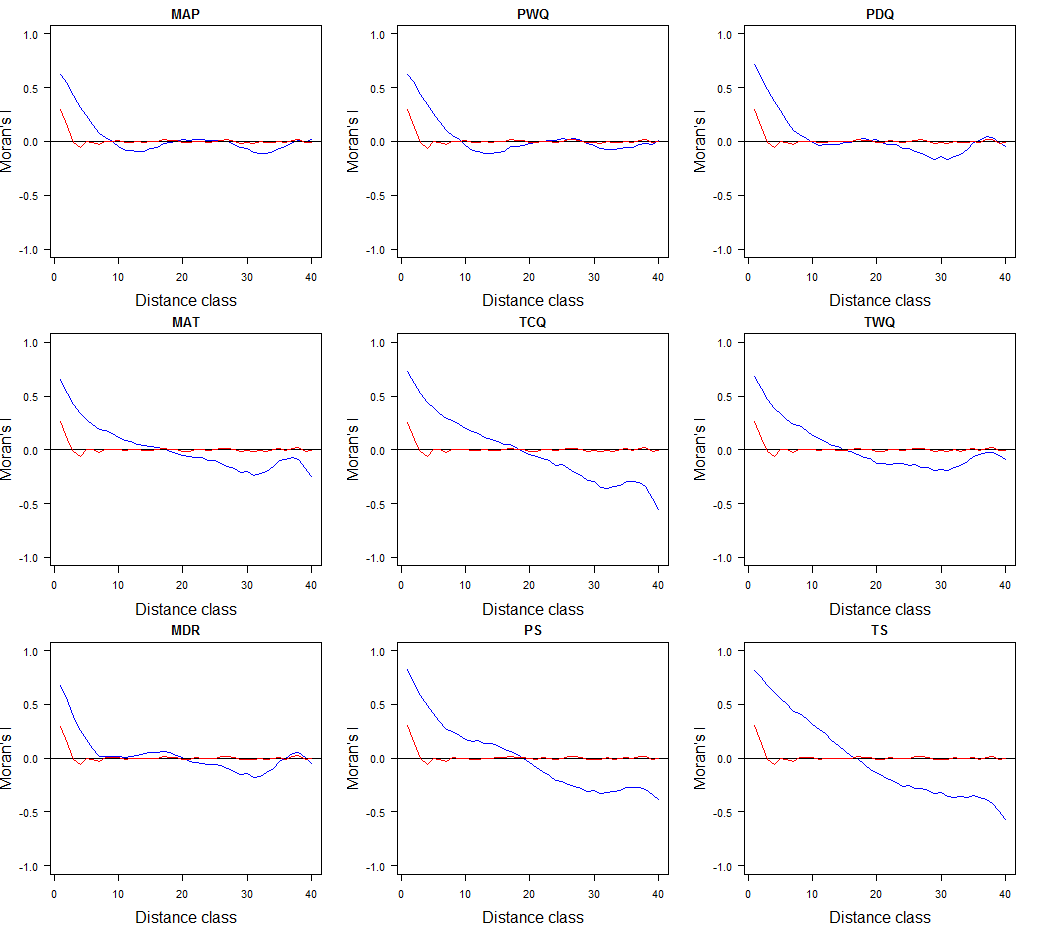


**Figure S6.** Moran's I correlograms for residuals of ordinary least squares regression models (blue) and residuals of spatial autoregressive error models (red) for herbaceous species. Both models included the proportion of fleshy-fruited species for herbaceous plants and the environment variables for MAP: mean annual precipitation; PWQ: precipitation of wettest quarter; PDQ: precipitation of driest quarter; MAT: mean annual temperature; TCQ: temperature of the coldest quarter; TWQ: mean temperature of warmest quarter; MDR: mean diurnal range; PS: precipitation seasonality; TS: temperature seasonality, respectively. The proportion of fleshy-fruited species was Log transformed in the analysis. All the precipitation variables were square root transformed before doing the analysis. One unit distance class corresponds to 100 km.
